# Apremilast for the treatment of hidradenitis suppurativa associated with psoriatic arthritis in multimorbid patients: Case report and review of literature

**DOI:** 10.1097/MD.0000000000018991

**Published:** 2020-01-31

**Authors:** Simone Garcovich, Giulia Giovanardi, Dalma Malvaso, Clara De Simone, Ketty Peris

**Affiliations:** Institute of Dermatology, Fondazione Policlinico Universitario A. Gemelli IRCCS, Università Cattolica del Sacro Cuore, Rome, Italy.

**Keywords:** apremilast, comorbidity, hidradenitis suppurativa, immune-mediated inflammatory disease, psoriatic arthritis

## Abstract

**Introduction:**

Hidradenitis suppurativa is a complex, chronic, difficult to treat condition belonging to the spectrum of cutaneous immune-mediated inflammatory diseases. Systemic treatment options for moderate-severe disease are limited to TNF-alpha antagonists and other biologic agents, with limited clinical evidence.

**Patient concerns:**

We report two adult patients with severe hidradenitis suppurativa presenting concomitant psoriatic arthritis and multiple medical comorbidities. Both were ineligible or resistant to adalimumab, the only biologic drug approved for the treatment of hidradenitis.

**Diagnosis:**

Both patients were diagnosed with severe Hurley III-stage disease and psoriatic arthritis, showing resistance to first-line systemic treatments and a complex comorbidity profile.

**Interventions:**

Patients underwent treatment with apremilast, an oral phosphodiesterase-4 inhibitor, approved for the treatment of psoriatic arthritis.

**Outcomes:**

After 16 weeks of treatment, a clinically relevant improvement of inflammatory lesions, skin- and arthritis-related pain, and patient-reported outcomes was achieved in both patients. Apremilast was well tolerated and continued up to 48 weeks of treatment.

**Conclusion:**

We report the “real-life” use of apremilast in the treatment of multimorbid patients with hidradenitis suppurativa and review its potential role in the management of this severe condition.

## 1 Introduction

Hidradenitis suppurativa (HS) is a complex, chronic inflammatory disorder of the follicular epithelium, presenting with recurrent, suppurative lesions at inverse body sites, such as axillary, inguinal, and anogenital regions. Like other immune-mediated inflammatory disorders (IMIDs), HS determines a profound impact on patient's quality of life and shares a significant association with cardiovascular and metabolic comorbidities. The treatment of HS is challenging and, currently, immune-modulating treatment is limited to adalimumab, the only officially approved drug for the treatment of moderate-severe disease.^[1]^ The lack of other approved immune-modulatory agents is especially frustrating in the case of patients with multiple comorbidities, resistance, or contraindications to TNF-alpha antagonists.

Recently, apremilast, a new class of oral-small molecules inhibiting phosphodiesterase-4, has been introduced in the treatment of IMIDs, such as plaque psoriasis and psoriatic arthritis (PsA).^[2]^ Preliminary clinical studies have also explored the use of apremilast in the treatment of moderate-severe, nonsyndromic HS, albeit with a low-comorbidity burden.

We report two patients with severe HS associated with PsA and a complex comorbidity profile, successfully treated with apremilast, and review the use of this new molecule in the management of HS.

## 2 Case report

### 2.1 Case 1

**Figure 1:**
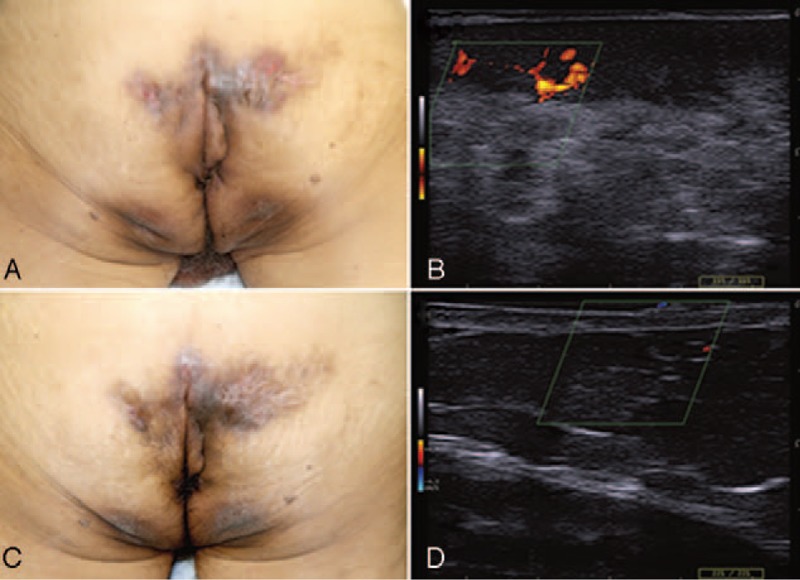
Case 1: (A) Severe Hurley III-hidradenitis suppurativa (HS-PGA 4/5) characterized by complex sinus-tracts and abscesses. (B) Doppler ultrasound examination showing inflamed tunnels on the right buttock, at baseline. (C) Clinical improvement after 16 weeks of apremilast treatment. (D) Decreased inflammation/color-flow signal after 16 weeks of treatment.

A 73-year-old man was presented with the diagnosis of HS since the age of 52 years, previously treated with oral antibiotics. At our observation, physical examination showed multiple fistulas and nodules on gluteal and anal region (Hurley III stage disease), associated with severe inflammation and pain. Clinical and patient-reported outcome measures included: the hidradenitis suppurativa-physician global assessment (HS-PGA = 4/severe), the numerical rating scale for pain (NRS-pain 7/10), the dermatology-life quality index (DLQI = 23), and C-reactive protein (CRP 4.8 mg/dl) (Fig. 1A and B). Ultrasound examination confirmed the presence of complex fistulas (Fig. 1B). In addition, concomitant PsA (DAPSA = 55), with a peripheral pattern of involvement, and localized plaque psoriasis (PASI = 5) were observed. The patient had a BMI = 21.72 and was a cigarette smoker (60 pack-years). He also had numerous comorbidities including NHYA class-III congestive heart disease, type-2 diabetes, iron deficiency anemia related to chronic bleeding from fistulas, and chronic renal insufficiency. Previous treatments included systemic antibiotics (tetracyclines, rifampicin, and clindamycin) for HS, and systemic steroids and NSAIDs for PsA. Based on the clinical profile and contraindication to TNF-alpha antagonists (congestive heart disease), treatment with apremilast (30 mg twice daily) was chosen. After 16 weeks of treatment, clinical and ultrasound examinations showed reduced inflammation and HS disease activity (HS-PGA = 2/mild disease) (Fig. 1C and D). Furthermore, a clinically relevant improvement of PsA (DAPSA = 12.8), inflammatory markers (CRP 2.8 mg/dl), and QoL (DLQI = 6) was achieved. The patient continued treatment with apremilast up to 40 weeks, also during non-HS-related surgical interventions for inguinal hernia. Extensive surgical treatment of gluteal skin lesions was not further indicated due to the complex comorbidity profile. At the last clinical follow-up (week 60), the patient was still on treatment with apremilast, without reporting any adverse event, and maintaining clinical remission of both PsA and HS disease.

### 2.2 Case 2

**Figure 2:**
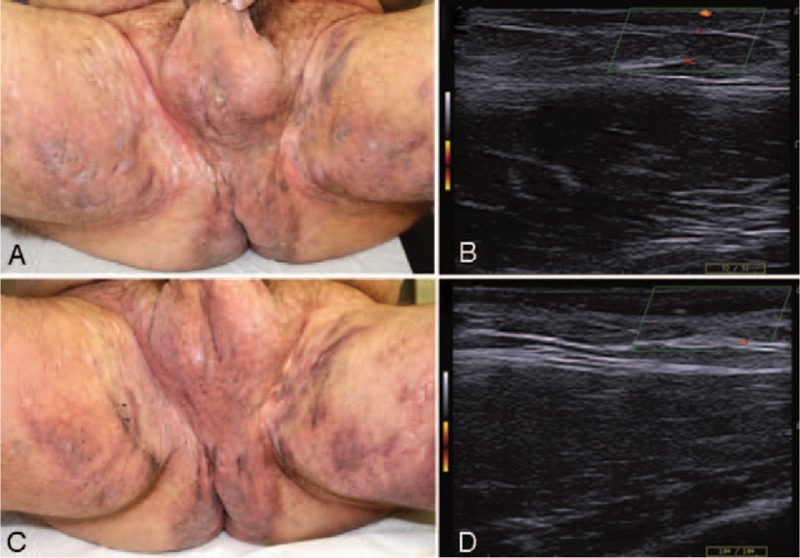
Case 2: (A) Very-severe Hurley III hidradenitis suppurativa (HS-PGA 5/5) with extensive involvement of the inguinal, perineal, perianal, and inner thigh areas. (B) Doppler ultrasound examination of left inguinal sinus-tract, at baseline. (C) Clinical improvement after 16 weeks of apremilast treatment. (D) Reduction of inflammation/color-flow signal, after 16 weeks of apremilast treatment.

A 51-year-old man was examined for a 7-year duration, severe HS (Hurley III) involving the groins and axillae. Physical examination showed multiple sinus-tracts and nodules on the pubic, inguinal, perianal, and axillary regions (HS-PGA = 5/very severe, NRS pain score 8/10, DLQI = 25) (Fig. 2A and B). The patient was also affected by localized plaque psoriasis and peripheral PsA (DAPSA = 48.2), type-2 diabetes, hypertension, nonalcoholic steatohepatitis, and nephrolithiasis. He smoked 30 pack-years and had BMI = 24. Previous therapies with systemic antibiotics (carbapenems, cotrimaxozole, beta-lactams), cyclosporine, corticosteroids, methotrexate, retinoids, and multiple biologic agents (infliximab, etanercept, adalimumab, secukinumab) were ineffective for both PsA and HS. Extensive surgical treatment of inguinal disease was postponed due to symptomatic nephrolithiasis and high inflammatory load (CRP 6.2 mg/dl). Due to lack of efficacy of previous treatments (adalimumab) and multiple comorbidities, treatment with apremilast (30 mg twice daily) was thus initiated. Apremilast determined a clinically relevant reduction of inflammatory lesions count, effective control of skin-related pain (NRS-pain score 0/10), and QoL-improvement (DLQI = 9) after 16 weeks of treatment (Fig. 2C and D). The course of HS clinical activity was consistent with disease stabilization (HS-PGA = 3/moderate), while PsA showed a marked reduction of disease activity (DAPSA = 16.7, CRP 2.7 mg/dl). Treatment with apremilast was further continued up to 48 weeks, with good tolerability, also during non-HS-related surgical interventions (nephrolithiasis). At the last clinical follow-up (week 72), the patient was still on active treatment with apremilast, with a stable disease course. The option of extensive surgical intervention was discussed with the multidisciplinary surgical team but later refused by the patient due to concern of surgery-associated morbidity.

## 3 Discussion

In both patients presented herein, the numerous comorbidities and concomitant IMID (psoriatic arthritis) posed a significant challenge for the medical management of moderate-severe HS. Other than adalimumab, the only FDA-approved biological drug, the clinical evidence to support the use of immune-modulating agents in HS is currently limited. In our cases, treatment with apremilast allowed to achieve a clinically relevant improvement of both IMIDs, HS-and PsA, and related patient-reported outcomes (pain, quality of life) after a period of 16 weeks. HS disease activity improved progressively in both patients, leading to disease stabilization of Hurley II-III disease and effective pain control. In the case of PsA, improvement of disease activity was more pronounced, as observed in clinical studies. Our patients also had numerous comorbidities that, however, did not impact the efficacy and safety of apremilast during the follow-up period. The patients did not report any adverse effect related to apremilast, nor abnormal laboratory examinations and both could undergo non-HS related surgical procedures, without interrupting treatment. The use of apremilast in the treatment of HS has been recently reported in two pilot clinical studies and in one case series, as summarized in Table 1, with a total of 59 cases of HS receiving apremilast (30 mg twice daily) for up to 36 weeks.^[3–5]^ Vossen et al reported the efficacy and tolerability of treatment with apremilast for 16 weeks in a small, double-blind randomized clinical trial, observing a significant clinical response (defined as a 50% improvement of inflammatory lesions count) in 53.3% of patients with moderate-severe disease.^[3]^ In another open-label, noncontrolled study, treatment with apremilast determined a significant clinical response in 60% of patients with mild-to-moderate HS after 24 weeks.^[4]^ An additional case-series of moderate-severe HS patients, who had responded poorly to other treatments, were treated with apremilast for up to 36 weeks, reporting a significant clinical response in five out of nine patients.^[5]^ None of the patients treated in previous studies presented other concomitant IMIDs or a relevant comorbidity burden. The adverse event profile of apremilast in HS patients seem to be consistent with the clinical data available in psoriasis patients, with a majority of mild-moderate events being gastro-intestinal symptoms and depression.^[2]^ Surprisingly, a recent ex-vivo study of lesional HS skin described only a limited effect of apremilast on the expression of key-inflammatory mediators, such as IL-17A, IL-17F cytokines, and S100A12 protein.^[6]^ On the other hand, the clinical evidence supports the role of apremilast in the management of moderate HS (Hurley stage II), especially to reduce inflammatory lesion count and to control disease activity in the long-term. Severe cases (Hurley stage III) patients will probably benefit more from potent, targeted anti-inflammatory treatment regimens in combination with extensive surgery. Other advantages of apremilast include no requirements for laboratory monitoring and a convenient dosing-scheme, which do not require dose-adjustments in patients with high BMI, a typical condition in HS. Combination treatment strategies with apremilast and other systemic agents should consider the risk for drug interactions with strong cytochrome-inducers, such as rifampicin, an antibiotic frequently used in HS.^[7]^

**Table 1:**
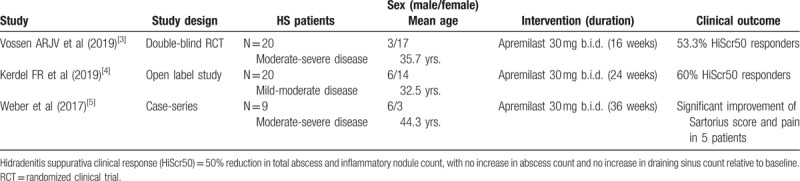
Summary of clinical studies of apremilast for the treatment of nonsyndromic hidradenitis suppurativa.

In conclusion, we report the use of apremilast in two patients with severe HS and PsA associated with a complex comorbidity profile. Management of multimorbid adult and elderly HS patients will pose a significant challenge in the near future, as observed in the aging psoriatic patient population.^[8]^ Considering the good safety profile as well as the low-risk of drug interactions, future controlled studies, with adequate sample sizes, should further explore the role of apremilast in the clinical management of hidradenitis suppurativa.

## Author contributions

**Conceptualization:** Simone Garcovich, Clara De Simone.

**Data curation:** Simone Garcovich, Giulia Giovanardi, Dalma Malvaso.

**Formal analysis:** Giulia Giovanardi.

**Supervision:** Clara De Simone, Ketty Peris.

**Validation:** Clara De Simone, Ketty Peris.

**Writing – original draft:** Simone Garcovich.

**Writing – review & editing:** Ketty Peris.

Simone Garcovich orcid: 0000-0001-8967-6688.
